# Body Mass Index and Its Association With Clinical and Pathological Features of Papillary Thyroid Carcinoma: A Retrospective Analysis

**DOI:** 10.7759/cureus.103518

**Published:** 2026-02-13

**Authors:** Khawla Salhi, Zineb Eddebbarh, Mohammed Amine Essafi, Zineb Elazime, Hayat Aynaou, Nadia Ismaili Alaoui, Houda Salhi

**Affiliations:** 1 Department of Endocrinology, Diabetology, Metabolic Diseases and Nutrition, Hassan II University Hospital, Fez, MAR; 2 Department of Endocrinology, Diabetology, Metabolic Diseases and Nutrition, Hassan II University Hospital Center, Fez, MAR; 3 Department of Nuclear Medicine, Hassan II University Hospital, Fez, MAR

**Keywords:** agressive histological outcomes, bmi, obesity, papillary thyroid carcinoma, pathology

## Abstract

Introduction

Papillary thyroid carcinoma (PTC) is a differentiated thyroid tumor. Its association with obesity and high body mass index has been suggested*.* This study investigates the potential link between elevated body mass index and aggressive features of PTC.

Materials and methods

A descriptive and analytical retrospective study was conducted on patients followed between January 2020 and January 2025 at the Nuclear Medicine and Endocrinology and Metabolic Diseases departments of Centre Hospitalier Universitaire (CHU; University Hospital Center) Hassan II in Fez. The inclusion criteria were patients >18 years old with PTC only. Patients with other histological types or conditions interfering with BMI were excluded. Epidemiological, clinical, pathological, and follow-up data were systematically collected. Data were compiled and analyzed using Microsoft Excel software (Microsoft Corporation, Redmond, USA).

Results

We included 243 cases: 78 men (25.4%) and 165 women (74.6%)*.* The mean age was 45 ± 12 years. The median BMI was 29.4 kg/m2, with 95 patients (39.09%) being overweight and 104 (42.7%) being obese. Postoperative anatomopathological analysis revealed a mean tumor size of 26.4 mm and a mean number of 9 ± 5 removed lymph nodes (LNs). Extrathyroidal invasion was found in 149 cases (61.3%), vascular invasion in 114 cases (46.9%), multifocal PTC in 59 cases (24.27%), cervical LN metastasis in 42 cases (17.28%), and extra-cervical metastasis in 17 patients (6.9%). A total of 81 cases (33.3%) presented with tumor-node-metastasis (TNM) stage III or IV. Statistical analysis showed a significant association between high BMI, female sex, and tumor size (p<0.05).

Conclusion

Our study shows that high BMI is correlated with some aggressive histological outcomes of PTC. Further studies are required to establish this impact in order to guide treatment and have favorable outcomes.

## Introduction

The most common type of differentiated thyroid tumors is the papillary thyroid carcinoma (PTC) [1]. Its incidence has been steadily increasing worldwide according to many recent studies; for example, in the United States, it increased by 3% annually from 1974 to 2013 [2]. The prevalence of obesity - and its complications - has also increased in the world [3], and it has a clear impact on cancer pathophysiology [4]. The impact of high body mass index (BMI) on the clinicopathological features of PTC has been reported in several studies recently [1]. The present study aims to determine whether increased BMI correlates with aggressive pathological features (age, sex, tumor size, multifocality, thyroiditis, lymph nodes, metastasis) in PTC or not.

## Materials and methods

We collected data retrospectively from 553 patients who had been diagnosed with PTC after thyroidectomy. These patients were followed up at the Department of Nuclear Medicine, University Hospital Center Hassan II of Fez, Morocco, between January 2020 and January 2025. All patients provided informed consent and were made aware of the study.

Inclusion criteria

Patients over 18 years old, with complete demographic and clinical data, who were diagnosed with PTC only (confirmed through surgical and histopathological examination) and showed good compliance with treatment and follow-up, were included in the study.

Exclusion criteria

Patients younger than 18 years, who had other types of malignant tumors alongside PTC, suffered from chronic diseases or were on treatments that could affect their BMI, had been overweight or obese since childhood, showed poor compliance with levothyroxine treatment (which led to high TSH levels and weight gain), or did not give consent to participate, were excluded from the study.

After applying these criteria, we narrowed down our study population from 553 to 243 patients.

We categorized BMI as follows: underweight (BMI ≤18.5 kg/m²), normal weight (BMI between 18.5 and 24.9 kg/m²), overweight (BMI between 25 and 29.9 kg/m²), and obese (BMI ≥30 kg/m²). We defined multifocality as having more than one PTC lesion in a single thyroid lobe.

We gathered information on age, gender, height, weight, thyroiditis status, tumor size, multifocality, bilaterality, metastasis, and other relevant clinical and pathological factors.

For each patient, we calculated BMI at their first clinical visit using their weight in kilograms divided by their height in meters squared. We assessed tumor size, thyroid volume, and multifocality through imaging studies such as ultrasound, CT scans, and MRI, as well as through examination of surgical specimens. Tumor measurements were recorded in cubic centimeters. We diagnosed thyroiditis based on clinical symptoms, lab tests, and imaging results, which were then confirmed by examining surgical specimens for inflammatory changes under the microscope. We detected metastasis using ultrasound, CT scans, MRI, scintigraphy, and by examining lymph nodes during surgery. A team of consultants reviewed all assessments to reduce potential bias.

We divided our patients into two groups: those who were overweight or obese, and those who had a normal weight or were underweight. We then compared various clinical and pathological characteristics between these groups, including age, gender, tumor size, multifocality, and lymph node metastasis.

We analyzed all the clinical and pathological data (age, sex, tumor size, multifocality, thyroiditis, lymph node involvement, and metastasis) using SPSS version 25.0 statistical software (IBM Corp., Armonk, USA). We considered results statistically significant when the P value was less than 0.05.

**Figure 1:**
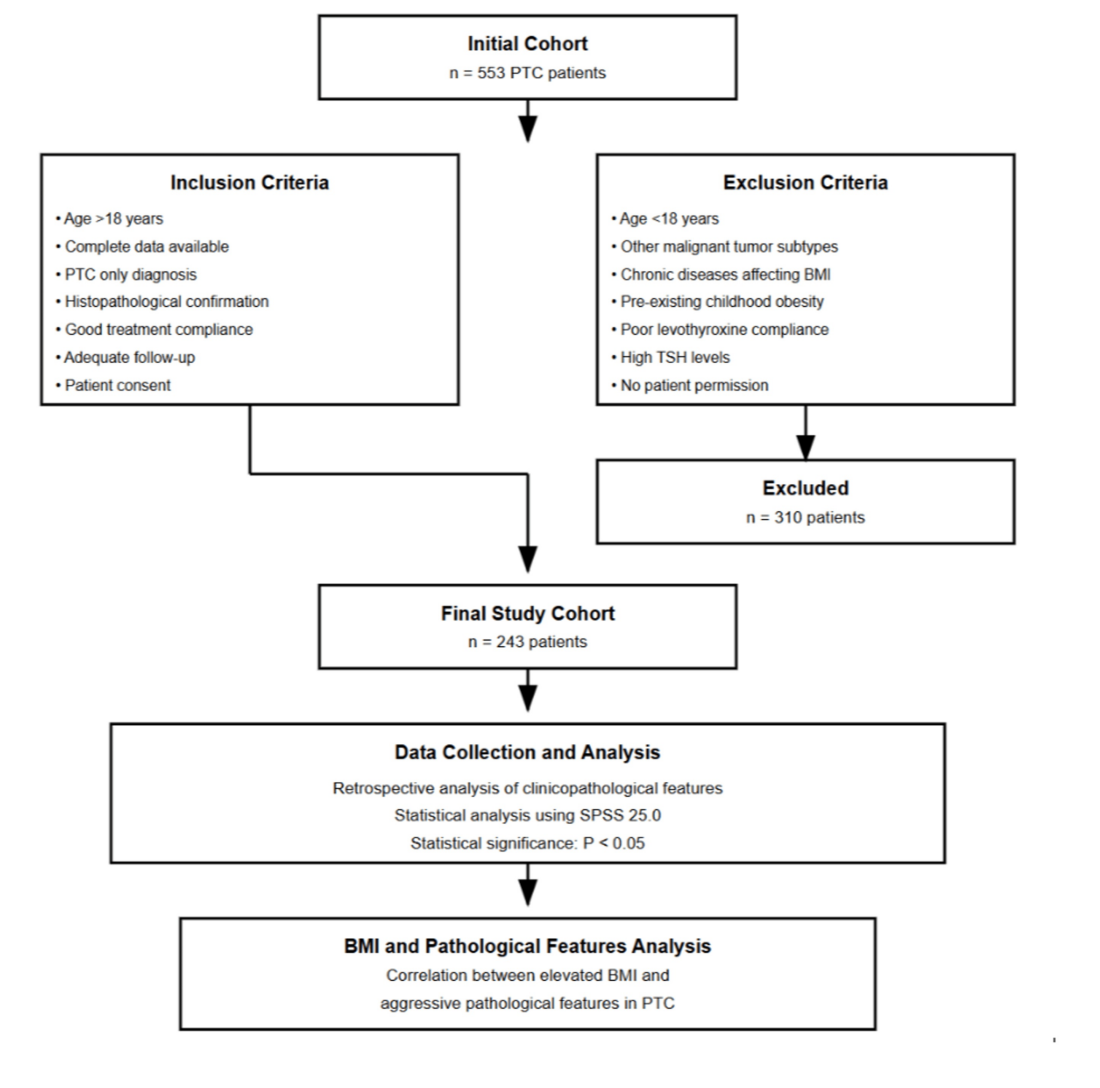
Flow chart of the study PTC: papillary thyroid carcinoma; TSH: thyroid-stimulating hormone SPSS statistical software by IBM Corp., Armonk, USA.

The flow chart of the study is illustrated in Figure 1.

## Results

We enrolled 243 patients who met our inclusion criteria (Figure 1). Of these, 78 were men (25.4%), and 165 were women (74.6%). The mean age of the patients was 45 ± 12 years (Table 1).

**Table 1:** Baseline characteristics of patients with papillary thyroid carcinoma (n = 243)

Characteristic	Value
Total patients, n	243
Gender, n (%)	
Male	78 (32.1%)
Female	165 (67.9%)
Age, years (mean ± SD)	45 ± 12

**Figure 2:**
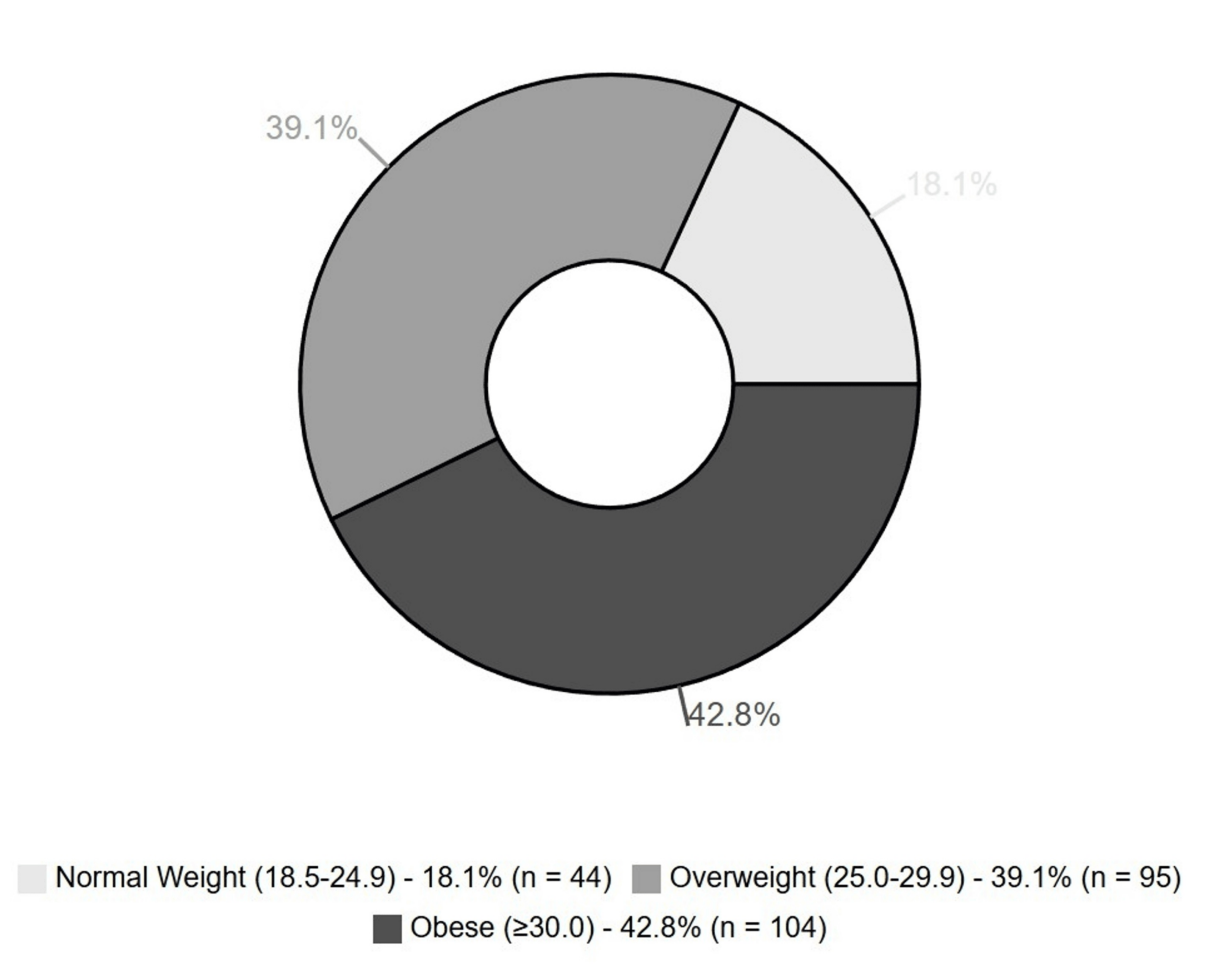
Distribution of BMI categories among patients with papillary thyroid carcinoma BMI range in parentheses in kg/m2.

The median BMI was 29.4 kg/m2 (range: 20-66 kg/m2), with 95 (39.09%) as overweight and 104 (42.7%) as obese patients (Figure 2). 

**Figure 3:**
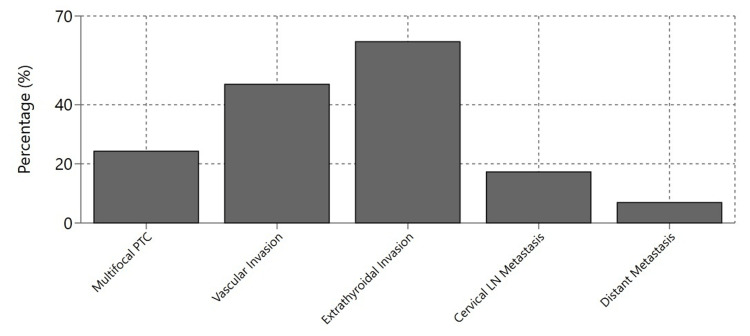
Distribution of pathological findings Values: Extrathyroidal invasion (61.3%), Vascular invasion (46.9%), Multifocal PTC (24.27%), Cervical LN metastasis (17.28%), Distant metastasis (6.9%) PTC: papillary thyroid carcinoma; LN: lymph node

The postoperative anatomopathological examination showed a mean tumor size of 26.4 mm and an average of 9 ± 5 lymph nodes removed (Table 2). Multifocal PTC was found in 59 cases (24.27%), vascular invasion in 114 cases (46.9%), and extrathyroidal invasion in 149 cases (61.3%) (Figure 3). Cervical lymph node metastasis was present in 42 patients (17.28%), while extra-cervical metastasis was found in 17 patients (6.9%) (Table 3). A total of 81 patients (33.3%) presented with tumor-node-metastasis (TNM) stage III or IV disease.

**Table 2:** Tumor characteristics

Parameter	Value
Mean tumor size (mm)	26.4
Mean number of removed lymph nodes (mean ± SD)	9 ± 5
Total cases	243

**Table 3:** Positive findings summary (n = 243) PTC: papillary thyroid carcinoma; LN: lymph node

Pathological Finding	Positive Cases	Percentage (%)
Multifocal PTC	59	24.27
Vascular Invasion	114	46.9
Extrathyroidal Invasion	149	61.3
Cervical LN Metastasis	42	17.28
Distant Metastasis	17	6.9

The mean age was 41 ± 7 years in the overweight group and 44 ± 3 years in the obese group. Among overweight patients, 27.7% were male, while only 4.7% of obese patients were male. The mean tumor size was 18.9 mm in overweight patients and 29.2 mm in obese patients. Multifocal lesions were found in 22.2% of overweight patients compared to 34.5% of obese patients. Thyroiditis was present in 16.8% of overweight patients and 25.8% of obese patients. Lymph node metastasis was found in 17.6% of overweight patients versus 21.4% of obese patients (Table 4).

**Table 4:** Clinicopathological characteristics by BMI groups

Clinicopathological Factor	Overweight Group (n=95)	Obese Group (n=104)	P-value
Age, mean ± SD (years)	41 ± 7	44 ± 3	0.127
Male gender, n (%)	26 (27.7%)	5 (4.7%)	0.003
Mean tumor size (mm)	18.9	29.2	0.021
Multifocal lesions, n (%)	21 (22.2%)	36 (34.5%)	0.084
Thyroiditis, n (%)	16 (16.8%)	27 (25.8%)	0.192
Lymph node metastasis, n (%)	17 (17.6%)	22 (21.4%)	0.521

Among PTC patients, overweight and obesity were significantly associated with female sex and tumor size (p<0.05), whereas no significant correlations were identified with age, Hashimoto's thyroiditis, or metastatic status.

## Discussion

The most common endocrine cancer is thyroid cancer (TC). Its incidence has been increasing recently. This increase is partly linked to the development of diagnostic tools, and partly to many other factors, including iodine intake, radiation exposure, imaging with iodine-containing media, eating habits, obesity, and exposure to endocrine disruptors [5,6]. Around 75-85% of TC is a PTC that is described as a well-differentiated cancer, growing slowly, with an overall survival rate of 96.6% [2]. Obesity is a public health issue due to its morbidity and mortality [7]. According to some recent studies, there is a positive association between high BMI and the risk of occurrence of many types of cancer, including thyroid cancer [8,9]. The association between high BMI and PTC has been widely discussed in the past few years [1]. 

Many hypotheses have been suggested to explain the link between obesity and TC: insulin resistance (IR) observed in obesity promotes signaling of thyroid-stimulating hormone (TSH) and insulin-like growth factor-1 (IGF-1), inflammation, and oxidative stress [5]. The synthesis of IGF-binding proteins (IGFBPs) is decreased in hyperinsulinemia, which leads to an increase in free circulating IGF-1. Both Insulin and IGF-1 have mitogenic abilities, and high IGF-1 levels accelerate cell proliferation in vitro. Insulin growth factor receptors are overexpressed in thyroid cancer cells compared to normal cells [10]. Several studies show that adiponectin, an anti-tumor agent, is low in high BMI cases. Another hypothesis was in relation to high TSH levels and hypothyroidism; for this reason, many recent studies excluded abnormal TSH from their study criteria [2]. 

Body mass index (BMI) may significantly influence anatomical considerations in clinical assessments. Patients with elevated BMI might experience physical characteristics that complicate both clinical examinations and self-screening for potential abnormalities. Moreover, increased adiposity could potentially compromise the diagnostic precision of imaging modalities, such as ultrasonography, leading to delayed diagnostic identification and more advanced disease progression. Multiple studies consistently demonstrated a notable correlation between elevated BMI and more advanced TNM cancer staging. While high BMI was linked to increased tumor size across overweight and obese patients, as our study shows. Economides et al's meta-analysis revealed that lymph node metastasis demonstrated an association with higher BMI in both overweight and obese patient groups, though statistical significance was restricted to the obesity subgroup [2].

O'Neill et al's meta-analysis demonstrated that obesity correlates with several critical tumor parameters, including increased tumor size, multifocality, extrathyroidal extension, and lymph node metastasis. These associations appeared more pronounced with progressive BMI increments. Notably, their findings did not establish significant connections between obesity and vascular invasion or overall patient outcome [1]. 

Conversely, Feng et al proposed that obesity in PTC patients specifically relates to extrathyroidal and vascular invasion. While acknowledging obesity's association with aggressive clinicopathological features, they cautiously concluded that obesity does not significantly influence postoperative complication rates or local recurrence risks [11]. 

Divergent perspectives emerged from Li et al, who argued that obesity correlates with LN metastasis, which potentially worsens patient outcomes [12]. In contrast, Gąsior-Perczak et al maintained that obesity neither predicts more aggressive tumor characteristics nor serves as a reliable prognostic indicator for disease progression or treatment response [13]. 

For our study, a strong correlation between overweight/obesity, female patients, and tumor size was established, but no clear impact has been shown on aggressive pathological features or prognosis. 

These inconsistent research findings underscore the complexity of understanding obesity's multifaceted role in thyroid cancer pathology, highlighting the critical need for further comprehensive and standardized investigations [14]. 

This study has noteworthy strengths. We examined a considerable cohort of 243 patients, applying rigorous inclusion and exclusion criteria that enhanced data quality by eliminating potential confounders. Our comprehensive data collection enabled us to identify significant associations between elevated BMI, female gender, and larger tumor size, adding meaningful evidence to the literature on metabolic factors in thyroid cancer pathogenesis. The limitations of our study include: it is a single-center and retrospective study, does not include the evolution of obesity over time, or further body mass details like muscle mass, body fat, or circumference. The retrospective design introduces potential selection bias, as patients were not randomly selected and may not be representative of the broader population. Additionally, measurement bias could have occurred due to variations in data collection methods and the accuracy of recorded measurements across different time periods. The follow-up period for patients was short, not allowing for the study of the real impact on prognosis.

## Conclusions

Obesity represents a significant public health problem with established associations for various cancers, such as PTC. While current literature demonstrates a relationship between obesity and potentially aggressive histologic features, the clinical significance remains incompletely understood. Our study demonstrates an association between elevated BMI and increased tumor size; however, no conclusive link with aggressive features such as lymph node metastasis or extrathyroidal extension was established. These findings suggest that the relationship between obesity and PTC aggressiveness may be more complex than initially hypothesized. We need multicenter, large-scale, prospective studies with standardized measures to establish these associations and elucidate the underlying biological mechanisms. Understanding these relationships will be crucial for developing evidence-based screening protocols and treatment strategies.
